# Supplementary material for: Characterization of mangrove mudflat sediment contamination by fecal bacteria and trace metals: A multivariate assessment in equatorial marine protected areas in Gabon, Western Central Africa
Source: PLoS One. 2025 Jun 17;20(6):e0326172. doi: 10.1371/journal.pone.0326172 (PMC12173363; doi:10.1371/journal.pone.0326172)
Supplement: S1 File — S1 Table. Sampling locations. Three-digit codes were designed according to local names (in French). Environment type and ESPG 4326 – WGS 84 coordinates are provided for each sampling location. S2 Table. Sampling schedule during the survey. ANP: Akanda; LBV: Libreville; PNP: Pongara National Park; sampling location codes are reported in S1 Table and figured in Fig 1. of the main manuscript. S3 Table. Sediment properties. Sediment in situ temperature and physico-chemical properties. S4 Table. Markers of biological pollution. Mean Fecal indicator bacteria (FIB) counts are given as colony-forming units (CFU), and mean chlorophyll a density in mg.m-2 for each sampling site during the whole survey. S5 Table. Trace metal concentrations in the sediments. The six trace metals retained for pollution assessment are Arsenic (As), Cadmium (Cd), Chromium (Cr), Copper (Cu), Lead (Pb) and Zinc (Zn), concentrations are given in µg.g-1 (dry weight of sediment). Iron (Fe) content in in µg.g-1 (dry weight of sediment) is reported as reference metal for Enrichment Factor determination. S6 Table. Concentration of elements quantified by ICP-MS, not included in assessment. Concentrations are given in µg.g-1 (dry weight of sediment). (ZIP) [file pone.0326172.s001.zip › S4_Table.docx]

# Characterization of mangrove mudflat sediment contamination by fecal bacteria and trace metals: A multivariate assessment in equatorial marine protected areas in Gabon – Supporting Information

*Contamination of mangrove mudflats in marine protected areas by urban sources*

Johann Ludovic Martial Happi ^1,2^, Aimé Roger Nzigou ^1^, Gauthier Schaal^3^, Marie-Laure Rouget^4^, Rolf Gael Mabicka Obame ^1^, François Le Loc’h^3^, Jean-Daniel Mbega ^5^, Christophe Leboulanger ^2^, Patrick Mickala ^1^

1. Université des Sciences et Techniques de Masuku, Franceville, Gabon
2. MARBEC, Univ Montpellier, CNRS, Ifremer, IRD, Sète, France
3. Univ Brest, CNRS, IRD, Ifremer, LEMAR, Plouzané, France
4. Institut Universitaire Européen de la Mer, IUEM, UAR 3113, Université de Bretagne Occidentale, CNRS, IRD, Plouzané, France
5. Laboratoire d’Hydrobiologie et d’Ichtyologie, IRAF, CENAREST, Libreville, Gabon

**S4 Table. Markers of biological pollution.** Mean Fecal indicator bacteria (FIB) counts are given as colony-forming units (CFU), and mean chlorophyll *a* density in mg.m^-2^ for each sampling site during the whole survey.

| **Sites** | **Stations** | ***E. coli* ± sd**  **(UFC/100 ml)** | **Strept ± sd**  **(UFC/100 ml)** | **Chl *a* ± sd**  **(mg/m^2^)** |
| --- | --- | --- | --- | --- |
| **LBV** | ALM | 0.00 | 325.25 ± 300.17 | 50.44 ± 24.17 |
|  | BBC | 20.8 ± 36.08 | 1066.67 ± 175.59 | 72.09 ± 26.74 |
|  | CCV | 0.00 | 281.25 ± 238.65 | 29.77 ± 26.62 |
|  | IG1 | 0.00 | 5845.83 ± 5062.64 | 35.63 ± 17.25 |
|  | IG2 | 275.00 ± 454.84 | 3050.00 ± 5239.45 | 92.45 ± 52.66 |
|  | LW1 | 21.50 ± 30.41 | 5025.00 ± 7035.71 | 56.57 ± 22.16 |
|  | LW2 | 0.00 | 2648.75 ± 4902.43 | 100.76 ± 22.13 |
|  | LW3 | 17.50 ± 35.00 | 2114.38 ± 3894.26 | 19.75 ± 6.15 |
|  | TMG | 150.00 ± 132.29 | 904.17 ± 956.09 | 54.31 ± 31.71 |
| **ANP** | KEN | 50.00 ± 86.60 | 587.67 ± 601.96 | 41.03 ± 16.74 |
|  | MAM | 0.00 | 370.83 ± 332.21 | 28.40 ± 15.83 |
|  | MKC | 0.00 | 1558.50 ± 1112.68 | 21.28 ± 4.72 |
|  | MKE | 0.00 | 891.83 ± 911.01 | 46.83 ± 13.12 |
|  | MKP | 208.33 ± 360.84 | 883.50 ± 133.95 | 68.19 ± 16.78 |
|  | NDE | 45.83 ± 79.39 | 237.50 ± 97.63 | 33.77 ± 17.37 |
|  | NZM | 50.00 ± 86.60 | 187.50 ± 120.55 | 31.68 ± 13.47 |
|  | PAG | 0.00 | 920.83 ± 356.71 | 45.21 ± 13.53 |
| **PNP** | PG1 | 0.00 | 0.00 | 30.74 ± 2.42 |
|  | PG2 | 0.00 | 2950.00 ± 4171.93 | 42.71 ± 16.19 |
|  | PG3 | 2.00 | 10000.00 | 41.94 |
|  | PG4 | 0.00 | 1388.00 | 54.21 ± 32.46 |
|  | PG5 | 0.00 | 10000.00 | 57.27 |
|  | PG6 | 0.00 | 6666.67 ± 5773.50 | 48.92 ± 15.37 |
|  | PG7 | 35.00 | 8500.00 | 100.13 |
