# Supplementary material for: Characterization of mangrove mudflat sediment contamination by fecal bacteria and trace metals: A multivariate assessment in equatorial marine protected areas in Gabon, Western Central Africa
Source: PLoS One. 2025 Jun 17;20(6):e0326172. doi: 10.1371/journal.pone.0326172 (PMC12173363; doi:10.1371/journal.pone.0326172)
Supplement: Attachment — Submitted filename: Plos one review comments.docx [file pone.0326172.s002.docx]

**Article: Characterization of mangrove mudflat sediment contamination by fecal bacteria and trace metals: A multivariate assessment in equatorial marine protected areas in Gabon**

The manuscript presents a potentially valuable study on pollution levels in mangroves within Gabon. However, to enhance its impact and accessibility, I recommend revisions addressing the contextual information provided, the justification for certain conclusions, and the overall flow of the narrative.

**Specific Comments:**

- I strongly suggest modifying the title to include geographical identifiers beyond just "Gabon." Specifying "Gabon, Africa" or "Gabon, Central Africa" will immediately orient readers unfamiliar with the country's location. This small change will significantly broaden the audience who can quickly grasp the study's focus.
- The abstract should begin by establishing the broader significance of mangroves, their global distribution, and their critical role in coastal ecosystems. Quantify their area cover globally and within Gabon if possible. Furthermore, clearly articulate the vulnerability of mangroves to pollution, emphasizing the potential consequences for biodiversity and ecosystem services. Then, explicitly connect this overarching context to the specific research question and approach undertaken in the study. This will highlight the relevance and importance of your work.
- Introduce the primary anthropogenic threats faced by Gabon's mangrove ecosystems in the abstract. Mention specific activities like oil and gas extraction, urbanization, or deforestation, as applicable. Briefly explain how these threats might contribute to the observed pollution levels and how your findings inform potential mitigation strategies. This helps readers understand the real-world implications of your research.
- Expand upon the role of Marine Protected Areas (MPAs), specifically the MNP mentioned in the manuscript. Explain the rationale behind designating these areas as MPAs. Detail the presence of Endangered, Threatened, and Protected (ETP) species within the MNP, highlighting ecologically sensitive zones within the protected area. This provides crucial context for understanding the potential impacts of pollution on vulnerable species and habitats and underlines the need for targeted conservation efforts. Discuss the ecological significance of mangroves harboring diverse marine fauna and how pollutants might negatively impact them.
- **Justification for Conclusions on MPA Effectiveness (Lines 36-40):** The conclusion that MPAs are not effective based on the observed low contamination levels requires substantial justification. This statement seems premature and potentially misleading without further analysis. I recommend carefully re-evaluating this conclusion and providing a more nuanced interpretation. Consider the possibility that the existing contamination levels are *lower than they would be without* the MPAs, even if they are not zero. The existing MPA may contribute to preventing further contamination, which this experiment would not test. Acknowledge the limitations of the study design in assessing the effectiveness of the MPAs.
- Include a literature review of case studies where MPAs have demonstrably reduced pressure on mangrove tidal flats. If such studies exist, cite them to provide a broader context for your findings. If studies show limited effectiveness in some contexts, acknowledge these and discuss potential reasons, such as proximity to industrial discharge or inadequate enforcement of regulations. Provide details regarding the proximity of industrial discharge to the studied MNPs and the number of industries located nearby. This information is crucial for evaluating the potential sources of pollution and assessing the effectiveness of the MPAs.
- Similar to the abstract, the introduction should begin by introducing Gabon and emphasizing its significance, particularly in terms of its biodiversity, coastal ecosystems, and role in the regional economy. Provide key geographical and ecological information that is relevant to the study.
- Lines 52-53 mention important coastal wetlands. Provide more detailed information about these wetlands, either in a table or in the text. Include details like their size, ecological characteristics, and any existing conservation status.
- Expand the introduction to include specific information about Gabon's mangroves. Discuss their species diversity, area cover, and ecological importance. Link this information back to lines 52-53, highlighting the importance of these mangroves within the broader context of Gabon's coastal wetlands. Providing statistics and key facts about these mangrove ecosystems will significantly strengthen the introduction.

**Methodology:**

- **Relocation of Lines 102-113:** I agree that the information presented in lines 102-113 would be more appropriate within the introduction, as it provides background context relevant to the study site selection and environmental conditions.
- **Clarity on Data Collection Campaigns:** Rephrase the term "data collection campaigns" to provide a clearer explanation of the specific activities involved. Describe the frequency, duration, and specific parameters measured during these campaigns.
- **Table 4 Terminology:** Change "This work" to "Present study" in Table 4 for consistency and professionalism.

**Discussion and Conclusion:**

- **Management Recommendations:** The discussion and conclusion should include practical recommendations for management measures that can be implemented to protect Gabon's mangroves. These recommendations should be based on the observations and results of the present study. Consider suggesting specific actions related to pollution control, MPA management, or community engagement.

By addressing these points, the authors can enhance the clarity, context, and impact of their research. The revisions will not only make the manuscript more accessible to a broader audience but also strengthen the conclusions and provide valuable insights for mangrove conservation in Gabon. The research has the potential to contribute significantly to the understanding of anthropogenic impacts on coastal ecosystems and inform effective conservation strategies.
