# Supplementary material for: Characterization of mangrove mudflat sediment contamination by fecal bacteria and trace metals: A multivariate assessment in equatorial marine protected areas in Gabon, Western Central Africa
Source: PLoS One. 2025 Jun 17;20(6):e0326172. doi: 10.1371/journal.pone.0326172 (PMC12173363; doi:10.1371/journal.pone.0326172)
Supplement: Attachment — Submitted filename: PONE-D-25-11485_Rebuttal_letter.docx [file pone.0326172.s004.docx]

**Rebuttal letter**

Dear Editor, Dear Reviewers,

We warmly acknowledge your comments and suggestions. The manuscript has been carefully modified to ensure that all your input contributes to its interest, and that it will finally deserve acceptance for publication in PLOS One.

For convenience, our responses are highlighted in blue, in boxed paragraph, and reference to the revised manuscript line numbers provided accordingly.

**Editor’s comments:**

Journal Requirements:

When submitting your revision, we need you to address these additional requirements.

1. Please ensure that your manuscript meets PLOS ONE's style requirements, including those for file naming. The PLOS ONE style templates can be found at

https://journals.plos.org/plosone/s/file?id=wjVg/PLOSOne_formatting_sample_main_body.pdf and

https://journals.plos.org/plosone/s/file?id=ba62/PLOSOne_formatting_sample_title_authors_affiliations.pdf

Style was checked, namely section headings were modified, and “Fig.” replaced by “Fig” throughout the manuscript.

2. Thank you for stating the following financial disclosure:

“PhD stipend to Johann L.M. Happi by the ARTS program of IRD (French National Research Institute for Sustainable Development)”

Please state what role the funders took in the study. If the funders had no role, please state: 'The funders had no role in study design, data collection and analysis, decision to publish, or preparation of the manuscript.'

If this statement is not correct you must amend it as needed.

The financial disclosure was modified accordingly:

“Johann L.M. Happi benefited from a PhD stipend through the ARTS Program of IRD (French National Research Institute for Sustainable Development). The Funder had no role in study design, data collection and analysis, decision to publish, or preparation of the manuscript.“

Please include this amended Role of Funder statement in your cover letter; we will change the online submission form on your behalf.

3. Thank you for stating the following in the Acknowledgments Section of your manuscript:

“This research is part of a PhD thesis (JLMH) funded by the IRD ARTS scholarship program and conducted under CNAR agreements AR022/21 and AR043/22, granted by CENAREST and approved by the Agence Nationale des Parcs Nationaux (ANPN) (work permits AE22008 and AE220017), Republic of Gabon. The authors thank the Gabonese authorities for their administrative support, particularly National Park curators M. Gilbert Moukanga (Akanda NP), M. Luc Patrick Evezo’o (Pongara NP), and ANPN staff for their help with field sampling. Special thanks to Romain Gonzalvez for his help preparing samples for ICP-MS analysis, and to Professor Makaya M’voubou and Dr. Mokea-Niaty for their hospitality and assistance during granulometric and organic matter content analyses at the USTM polytechnic school and Biology Department laboratories.”

We note that you have provided funding information that is currently declared in your Funding Statement. However, funding information should not appear in the Acknowledgments section or other areas of your manuscript. We will only publish funding information present in the Funding Statement section of the online submission form.

Please remove any funding-related text from the manuscript and let us know how you would like to update your Funding Statement. Currently, your Funding Statement reads as follows:

“PhD stipend to Johann L.M. Happi by the ARTS program of IRD (French National Research Institute for Sustainable Development)”

Please include your amended statements within your cover letter; we will change the online submission form on your behalf.

Funding-related text was removed from the Acknowledgement section accordingly.

4. When completing the data availability statement of the submission form, you indicated that you will make your data available on acceptance. We strongly recommend all authors decide on a data sharing plan before acceptance, as the process can be lengthy and hold up publication timelines. Please note that, though access restrictions are acceptable now, your entire data will need to be made freely accessible if your manuscript is accepted for publication. This policy applies to all data except where public deposition would breach compliance with the protocol approved by your research ethics board. If you are unable to adhere to our open data policy, please kindly revise your statement to explain your reasoning and we will seek the editor's input on an exemption. Please be assured that, once you have provided your new statement, the assessment of your exemption will not hold up the peer review process.

All the data used are currently available without restriction in the Public Repository of the French Research Institute for Sustainable Development (https://dataverse.ird.fr/). Data were curated by the dedicated staff, provided with metadata and relevant information. The Digital Object Identifier address is: https://doi.org/10.23708/GN6XDK

Metadata will be completed with the full reference of the article after acceptance in PLOS One.

5. Please include your full ethics statement in the ‘Methods’ section of your manuscript file. In your statement, please include the full name of the IRB or ethics committee who approved or waived your study, as well as whether or not you obtained informed written or verbal consent. If consent was waived for your study, please include this information in your statement as well.

Ethics statement was provided in the ‘Methods’ section: the Gabonese National Committee for Research Authorizations (Comité National des Autorisations de Recherche – CNAR) is the relevant authority and serves as Institutional Review Board in Gabon. Accordingly, as some of the sampling was performed in protected areas, the Gabonese National Agency for National Parks (Agence Nationale des Parcs Nationaux – ANPN) provided specific permits.

The relevant section in the revised manuscript was added at the end of Methods section:

Ethics statement

The research authorization and the work permit in protected areas were granted respectively by CENAREST review board (Comité National des Autorisations de Recherche, CNAR) and the Scientific Unit of ANPN (Gabonese National Agency for National Parks). (Lines 257-260)

6. We note that Figure 1 in your submission contain [map/satellite] images which may be copyrighted. All PLOS content is published under the Creative Commons Attribution License (CC BY 4.0), which means that the manuscript, images, and Supporting Information files will be freely available online, and any third party is permitted to access, download, copy, distribute, and use these materials in any way, even commercially, with proper attribution. For these reasons, we cannot publish previously copyrighted maps or satellite images created using proprietary data, such as Google software (Google Maps, Street View, and Earth). For more information, see our copyright guidelines: http://journals.plos.org/plosone/s/licenses-and-copyright.

We require you to either (1) present written permission from the copyright holder to publish these figures specifically under the CC BY 4.0 license, or (2) remove the figures from your submission:

a. You may seek permission from the original copyright holder of Figure 1 to publish the content specifically under the CC BY 4.0 license.

We recommend that you contact the original copyright holder with the Content Permission Form (http://journals.plos.org/plosone/s/file?id=7c09/content-permission-form.pdf) and the following text:

“I request permission for the open-access journal PLOS ONE to publish XXX under the Creative Commons Attribution License (CCAL) CC BY 4.0 (http://creativecommons.org/licenses/by/4.0/). Please be aware that this license allows unrestricted use and distribution, even commercially, by third parties. Please reply and provide explicit written permission to publish XXX under a CC BY license and complete the attached form.”

Please upload the completed Content Permission Form or other proof of granted permissions as an ''Other'' file with your submission.

In the figure caption of the copyrighted figure, please include the following text: “Reprinted from [ref] under a CC BY license, with permission from [name of publisher], original copyright [original copyright year].”

b. If you are unable to obtain permission from the original copyright holder to publish these figures under the CC BY 4.0 license or if the copyright holder’s requirements are incompatible with the CC BY 4.0 license, please either i) remove the figure or ii) supply a replacement figure that complies with the CC BY 4.0 license. Please check copyright information on all replacement figures and update the figure caption with source information. If applicable, please specify in the figure caption text when a figure is similar but not identical to the original image and is therefore for illustrative purposes only.

The following resources for replacing copyrighted map figures may be helpful:

USGS National Map Viewer (public domain): http://viewer.nationalmap.gov/viewer/

The Gateway to Astronaut Photography of Earth (public domain): http://eol.jsc.nasa.gov/sseop/clickmap/

Maps at the CIA (public domain): https://www.cia.gov/library/publications/the-world-factbook/index.html and https://www.cia.gov/library/publications/cia-maps-publications/index.html

NASA Earth Observatory (public domain): http://earthobservatory.nasa.gov/

Landsat: http://landsat.visibleearth.nasa.gov/

USGS EROS (Earth Resources Observatory and Science (EROS) Center) (public domain): http://eros.usgs.gov/#

Natural Earth (public domain): http://www.naturalearthdata.com/

Response: the map provided as Fig 1 is a personal work of First Author JLHM using Qgis freeware, based on https://www.openstreetmap.org and https://marineregions.org, and completed with publicly available data issued from Gabonese laws. References are therefore added in the Reference list and stated in the Fig 1 legend. (Akanda National Park: République Gabonaise 2007, Décret n° 608/PR/MEFEPEPN; Pongara National Park: République Gabonaise 2007, Décret n° 618/PR/MEFEPEPN; Arboretum Raponda Walker: République Gabonaise 2012, Décret n°0460/PR/MEF; Marine Reserves: République Gabonaise 2017, Décret N° 00161/PR)

**Reviewers' comments:**

**Reviewer's Responses to Questions**

Comments to the Author

1. Is the manuscript technically sound, and do the data support the conclusions?

The manuscript must describe a technically sound piece of scientific research with data that supports the conclusions. Experiments must have been conducted rigorously, with appropriate controls, replication, and sample sizes. The conclusions must be drawn appropriately based on the data presented.

Reviewer #1: Partly

Reviewer #2: Yes

2. Has the statistical analysis been performed appropriately and rigorously?

Reviewer #1: Yes

Reviewer #2: Yes

3. Have the authors made all data underlying the findings in their manuscript fully available?

The PLOS Data policy requires authors to make all data underlying the findings described in their manuscript fully available without restriction, with rare exception (please refer to the Data Availability Statement in the manuscript PDF file). The data should be provided as part of the manuscript or its supporting information, or deposited to a public repository. For example, in addition to summary statistics, the data points behind means, medians and variance measures should be available. If there are restrictions on publicly sharing data—e.g. participant privacy or use of data from a third party—those must be specified.

Reviewer #1: Yes

Reviewer #2: Yes

4. Is the manuscript presented in an intelligible fashion and written in standard English?

PLOS ONE does not copyedit accepted manuscripts, so the language in submitted articles must be clear, correct, and unambiguous. Any typographical or grammatical errors should be corrected at revision, so please note any specific errors here.

Reviewer #1: No

Reviewer #2: Yes

5. Review Comments to the Author

Please use the space provided to explain your answers to the questions above. You may also include additional comments for the author, including concerns about dual publication, research ethics, or publication ethics. (Please upload your review as an attachment if it exceeds 20,000 characters)

**Reviewer #1:**

The manuscript presents a potentially valuable study on pollution levels in mangroves within Gabon. However, to enhance its impact and accessibility, I recommend revisions addressing the contextual information provided, the justification for certain conclusions, and the overall flow of the narrative.

Specific Comments:

• I strongly suggest modifying the title to include geographical identifiers beyond just "Gabon." Specifying "Gabon, Africa" or "Gabon, Central Africa" will immediately orient readers unfamiliar with the country's location. This small change will significantly broaden the audience who can quickly grasp the study's focus.

Many thanks for this suggestion enhancing the readership impact. The title was modified as:

“Characterization of mangrove mudflat sediment contamination by fecal bacteria and trace metals: A multivariate assessment in equatorial marine protected areas in Gabon, Western Central Africa” accordingly.

• The abstract should begin by establishing the broader significance of mangroves, their global distribution, and their critical role in coastal ecosystems. Quantify their area cover globally and within Gabon if possible. Furthermore, clearly articulate the vulnerability of mangroves to pollution, emphasizing the potential consequences for biodiversity and ecosystem services. Then, explicitly connect this overarching context to the specific research question and approach undertaken in the study. This will highlight the relevance and importance of your work.

Thank you for the suggestion. A revised version of the Abstract now reads at the beginning:

“Among the most productive ecosystems worldwide, mangroves contribute to global carbon sequestration and play a pivotal role for many species, including supporting the sustainable provision of intertropical fisheries resources. []” (Lines 29-31)

• Introduce the primary anthropogenic threats faced by Gabon's mangrove ecosystems in the abstract. Mention specific activities like oil and gas extraction, urbanization, or deforestation, as applicable. Briefly explain how these threats might contribute to the observed pollution levels and how your findings inform potential mitigation strategies. This helps readers understand the real-world implications of your research.

These concerns are highly relevant. To meet the constraints of the Abstract, local threats are summarized as follows:

“[] Despite the many essential ecosystem services they provide, mangrove ecosystems are facing increasing anthropogenic pressure, primarily because they develop in littoral areas where human activities are rapidly expanding, causing deforestation, urban extension and pollution. Mangroves cover over 1,700 km² of Gabon's coastline, stretching within protected zones or located alongside populated areas, raising concerns about the potential impact of pollution. This study assessed pollution levels in mangrove surface sediments from 24 stations in the capital city of Libreville and the two adjacent Marine Protected Areas (MPAs), Akanda and Pongara, where human impact has not been fully characterized. []” (Lines 31-38)

• Expand upon the role of Marine Protected Areas (MPAs), specifically the MNP mentioned in the manuscript. Explain the rationale behind designating these areas as MPAs. Detail the presence of Endangered, Threatened, and Protected (ETP) species within the MNP, highlighting ecologically sensitive zones within the protected area. This provides crucial context for understanding the potential impacts of pollution on vulnerable species and habitats and underlines the need for targeted conservation efforts. Discuss the ecological significance of mangroves harboring diverse marine fauna and how pollutants might negatively impact them.

Many thanks for the suggestion. Unfortunately, these topics cannot be in-depth detailed in the Abstract section, but are taken into account in the Introduction (Lines 65-73); emphasis on current pressures in the area is detailed Lines 73-80.

• Justification for Conclusions on MPA Effectiveness (Lines 36-40): The conclusion that MPAs are not effective based on the observed low contamination levels requires substantial justification. This statement seems premature and potentially misleading without further analysis. I recommend carefully re-evaluating this conclusion and providing a more nuanced interpretation. Consider the possibility that the existing contamination levels are lower than they would be without the MPAs, even if they are not zero. The existing MPA may contribute to preventing further contamination, which this experiment would not test. Acknowledge the limitations of the study design in assessing the effectiveness of the MPAs.

This remark is extremely relevant. We therefore modified the last sentence of the Abstract that now reads:

“This study provides a first assessment of pollution pressure on mangrove tidal flats within protected areas in Gabon, emphasizing that the success of MPAs could be enhanced through contamination monitoring to achieve long-term conservation goals.” (Lines 48-50)

• Include a literature review of case studies where MPAs have demonstrably reduced pressure on mangrove tidal flats. If such studies exist, cite them to provide a broader context for your findings. If studies show limited effectiveness in some contexts, acknowledge these and discuss potential reasons, such as proximity to industrial discharge or inadequate enforcement of regulations. Provide details regarding the proximity of industrial discharge to the studied MNPs and the number of industries located nearby. This information is crucial for evaluating the potential sources of pollution and assessing the effectiveness of the MPAs.

Many thanks for the suggestion. Reviewing literature about MPAs effectiveness against pollution pressure is out of scope for the present work, and not suitable for an Abstract. Nevertheless, we provided information in the Introduction section (Lines 65-80) section, and consistent references were added.

• Similar to the abstract, the introduction should begin by introducing Gabon and emphasizing its significance, particularly in terms of its biodiversity, coastal ecosystems, and role in the regional economy. Provide key geographical and ecological information that is relevant to the study.

This information was indeed added to the revised manuscript, mostly by taking into account all the above remarks and suggestions. To go further in the role regarding economy focusing on coastal ecosystems, we currently lack of a robust account in scientific literature, since there is a very scarce recent and relevant knowledge available. Several references were added to support these ideas.

• Lines 52-53 mention important coastal wetlands. Provide more detailed information about these wetlands, either in a table or in the text. Include details like their size, ecological characteristics, and any existing conservation status.

An information complement is provided Lines 62-70.

• Expand the introduction to include specific information about Gabon's mangroves. Discuss their species diversity, area cover, and ecological importance. Link this information back to lines 52-53, highlighting the importance of these mangroves within the broader context of Gabon's coastal wetlands. Providing statistics and key facts about these mangrove ecosystems will significantly strengthen the introduction.

Many thanks for these suggestions. All the above cited modifications, together with additional references, were made according to this demand.

Methodology:

• Relocation of Lines 102-113: I agree that the information presented in lines 102-113 would be more appropriate within the introduction, as it provides background context relevant to the study site selection and environmental conditions.

We totally agree with this comment. The information about study sites was therefore distributed in the Introduction section for general considerations, whereas information strictly relevant to sampling and field work kept in the Methods (Lines 125-136).

• Clarity on Data Collection Campaigns: Rephrase the term "data collection campaigns" to provide a clearer explanation of the specific activities involved. Describe the frequency, duration, and specific parameters measured during these campaigns.

We are not fully sure of the above recommendation. For clarity, the subsection title was changed to “Sampling procedures” (Line 143). The date of each survey is provided as supplementary information (S2 Table) and a change was made regarding in situ temperature measurement that was misplaced (Lines 150-151)

• Table 4 Terminology: Change "This work" to "Present study" in Table 4 for consistency and professionalism.

Many thanks for these suggestions. Table 4 was modified accordingly (Line 456).

Discussion and Conclusion:

• Management Recommendations: The discussion and conclusion should include practical recommendations for management measures that can be implemented to protect Gabon's mangroves. These recommendations should be based on the observations and results of the present study. Consider suggesting specific actions related to pollution control, MPA management, or community engagement.

By addressing these points, the authors can enhance the clarity, context, and impact of their research. The revisions will not only make the manuscript more accessible to a broader audience but also strengthen the conclusions and provide valuable insights for mangrove conservation in Gabon. The research has the potential to contribute significantly to the understanding of anthropogenic impacts on coastal ecosystems and inform effective conservation strategies.

Thank you for these suggestions. We are in full agreement with the reviewer, as the primary objective of this work is to establish an initial assessment of pollution pressures in our study area. However, in order to issue recommendations and propose specific actions, it is essential to be able to observe pollution trends over time, which requires a sustained and continuous evaluation of these pressures. Like many countries, Gabon currently lacks the full capacity to handle direct action, and we believe it would be premature for us to take on a prescriptive role. Nevertheless, we have addressed this comment by expanding the Discussion with contextual elements (Lines 489–494) and by strengthening the Conclusion section of the manuscript (Lines 709–722). We sincerely hope these efforts will help broaden the impact of this work.

Reviewer #2: Nice paper. Important work regarding mangrove pollution.

I have only identified unformatted text in the lines 412 and 413, item "4.3. Trace metals concentrations, contamination assessment and sources".

Thank you for your comment. Please be assured that your opinion is extremely gratifying to the first author, a doctoral student who has fully dedicated himself to his research subject.

The missing break was checked and fixed (Lines 435-436).

While revising your submission, please upload your figure files to the Preflight Analysis and Conversion Engine (PACE) digital diagnostic tool, https://pacev2.apexcovantage.com/. PACE helps ensure that figures meet PLOS requirements. To use PACE, you must first register as a user. Registration is free. Then, login and navigate to the UPLOAD tab, where you will find detailed instructions on how to use the tool. If you encounter any issues or have any questions when using PACE, please email PLOS at figures@plos.org. Please note that Supporting Information files do not need this step.

Please note that all Figures included in the submitted manuscript were processed through PACE system as requested, and were uploaded on the editoriamanager system without further changes.
